# Repurposing drugs to treat neurological diseases

**DOI:** 10.1007/s00415-018-8732-z

**Published:** 2018-01-10

**Authors:** T. H. Massey, N. P. Robertson

**Affiliations:** Division of Psychological Medicine and Clinical Neuroscience, Department of Neurology, Cardiff University, University Hospital of Wales, Heath Park, Cardiff, CF14 4XN UK

## Introduction

The development of new drugs can be expensive: on average, it costs over $2 billion to take a medication from inception to clinical practice. Drug discovery is particularly difficult in neuropsychiatric diseases as targets are often poorly defined, inaccessible, and not easily assayed. Furthermore, many neurological diseases progress slowly over many years, often requiring clinical trials examining efficacy to be large and have complex end points. However, one way of expediting drug development is to repurpose drugs from their original use to a new indication. Multiple examples exist in clinical medicine: sildenafil was an ineffective angina drug repurposed for erectile dysfunction; minoxidil was a hypertension drug repurposed for hair loss. These unexpected therapeutic discoveries were made serendipitously, and often as side-effects in clinical trials. Unbiased, high-throughput screens are now being used systematically to test libraries of clinically approved drugs in areas of medicine away from their usual indications. These screens can generate novel therapeutic avenues, shed light on molecular pathology, and lead directly to clinical trials.

This month’s journal club explores three papers relating to drug repurposing in Neurology. The first two papers use unbiased cell-based screens of drug/compound libraries to search for potential therapeutic activities relating to either Parkinson’s disease or Zika virus infection of neurons, respectively. The third paper reports a clinical trial of clemastine, commonly used as an antihistamine, in the remyelination of optic nerves in multiple sclerosis.

## β2-Adrenoreceptor is a regulator of the α-synuclein gene driving risk of Parkinson’s disease

Intraneuronal aggregates of phosphorylated α-synuclein (Lewy bodies) are found in the brains of patients with Parkinson’s disease (PD). Disease severity correlates with Lewy body formation, although there is debate about whether these aggregates are directly toxic to neurons. Overexpression of wild-type α-synuclein, either as a result of *SNCA* gene duplication/triplication in familial PD or from gene regulatory variants in sporadic disease, can drive PD. Furthermore, decreased α-synuclein production correlates with clinical improvement.

This paper reported a high-throughput screening assay for compounds that could reduce *SNCA* gene expression in human neuroblastoma cell lines. Cells were grown in multi-well plates, treated with one of 1126 drugs previously tested for safety in humans, and then harvested for gene expression analysis. Optimisation and replication of the screen ultimately identified four drugs that significantly reduced *SNCA* mRNA and protein levels in a dose-dependent manner: three β2-adrenoceptor (β2AR) agonists, including the common asthma medication salbutamol, and riluzole, a drug licensed in amyotrophic lateral sclerosis. Conversely, β2AR inhibition by propranolol, a non-specific β-blocker, led to increased *SNCA* mRNA and protein levels. Extending the observations to animal models, intraperitoneal administration of β2AR agonist clenbuterol to wild-type mice significantly reduced *SNCA* expression in the substantia nigra. Similar clenbuterol administration to a mouse model of PD prevented nigral neuronal loss.

Finally, the study took advantage of the Norwegian Prescription Database (NorPD) which contains all information about individual prescriptions in Norway since 2004. Interestingly, if an individual had ever used salbutamol (*n* = 619,863), they had a significantly lower incidence of PD (rate ratio = 0.66, 95% confidence interval 0.58–0.76), and, conversely, if they had ever used propranolol for at least 1 year (*n* = 14,794), they had a significantly increased risk of PD (rate ratio = 2.20, 95% confidence interval 1.62–3.00). Adjustments were made for age, sex, and level of education, as well as attempts to control for other potential confounders such as smoking and essential tremor.

*Comment.* This study used a well-designed unbiased screen to discover compounds that can regulate α-synuclein gene expression. Unexpectedly, these included β2AR agonists widely used as asthma medications. The correlation of β2AR agonist use and reduced incidence of PD in the large Norwegian population fitted in well with the cellular and animal work presented, although whether *SNCA* expression is the critical driver of pathology in sporadic PD is unknown. Further work is needed to elucidate molecular mechanisms, but this should not prevent clinical trials of β2AR agonists in PD being fast-tracked.

Mittal S et al (2017) Science 357: 891–898.

## Identification of small-molecule inhibitors of Zika virus infection and induced neural cell death via a drug repurposing screen

Zika virus (ZIKV) is a mosquito-borne pathogen most commonly associated with a mild illness of fever, rash, headache, and myalgia. However, recent outbreaks in the Pacific islands and Brazil have been associated with serious neurological sequelae, such as microcephaly in newborn infants and Guillain–Barré syndrome in adults. Although the molecular pathology of ZIKV infection is incompletely understood, recent work has shown that ZIKV can infect human neural progenitor cells (hNPCs), inducing caspase-3 and subsequent cell death. Such neuronal loss, also observed following ZIKV infection of brain organoids in vitro, is compatible with the observed link between ZIKV and microcephaly.

This study screened over 6000 pharmacologically active compounds for the inhibition of ZIKV-induced caspase-3 activation in human glioblastoma cells. A second, independent, screen was carried out with the same compounds in hNPCs. A total of 116 compounds inhibited caspase-3 activation in both screens, and these were taken forward to cytotoxicity testing. After elimination of cytotoxic drugs, two broad classes of active compounds were identified: those that directly prevented cell death, and those that inhibited ZIKV replication and indirectly prevented cell death. In the first group, emricasan, a pan-caspase inhibitor in phase 2 clinical trials for hepatitis C-related hepatic injury, was an effective inhibitor of cell death following ZIKV infection. However, it did not inhibit ZIKV replication. In the second group, two compounds strongly inhibited ZIKV replication in cells in a dose-dependent manner. These drugs were niclosamide, used for treating worm infections, and an investigational cyclin-dependent kinase inhibitor (CDKi). Combining emricasan and a CDKi in treatment of ZIKV-infected hNPCs or astrocytes led to additive inhibition of caspase-3 activity as well as improved cell viability.

*Comment.* The high-throughput screens used in this study led to the rapid identification of potential therapeutics for ZIKV infection. Pan-caspase inhibitors were, unsurprisingly, found to be protective against caspase-3 activation in ZIKV-infected cells. Given the importance of apoptosis in normal physiology, these drugs may not have utility in ZIKV treatment. In contrast, the discovery of antiviral activity associated with niclosamide, an approved drug for worm treatment, suggests that repurposing this medication for Zika, and potentially other, viral infections could be a fruitful therapeutic avenue. Niclosamide can be used in pregnancy and so has the potential to be protective against microcephaly.

Xu M et al (2016) Nat Med 22: 1101–1107.

## Clemastine fumarate as a remyelinating therapy for multiple sclerosis (ReBUILD): a randomised, controlled, double-blind, crossover trial

Multiple sclerosis (MS) is characterised by inflammation and demyelination of neurons in the central nervous system (CNS). Current disease-modifying therapies can reduce relapse rates, and may delay neuronal loss, but there is no evidence that they lead to remyelination of damaged CNS neurons. Previous functional drug screens identified clemastine, a sedating antihistamine available over-the-counter, as having remyelinating potential through its off-target antimuscarinic effects. Clemastine can cross the blood–brain barrier and is able to drive neuronal remyelination in cell and animal models of MS.

This paper reported a phase 2 crossover trial of clemastine and placebo in patients with relapsing–remitting MS and chronic demyelinating optic neuropathy on stable immunomodulatory treatment (ReBUILD). Fifty patients were randomised 1:1 to either 3 months of clemastine followed by 2 months of placebo, or 3 months of placebo followed by 2 months of clemastine. The two groups were age- and disability-matched, but there were some differences in sex ratio and time since optic neuritis. Forty-six of the fifty patients were established on a variety of immunomodulatory therapies, although the drugs taken by the two groups were not reported. The primary outcome of the trial was shortening of P100 latency delay on visual-evoked potentials (VEPs), an indirect readout of optic nerve remyelination. Patients in both groups showed improved (shortened) P100 latencies on VEPs when taking clemastine, and this effect was sustained through the placebo period when clemastine was taken first. The improvements were small but statistically significant (mean improvement of 1.7 ms/eye, 95% confidence interval 0.5–2.9 ms, *p* = 0.0048, baseline P100 latency > 118 ms). A small, correlated improvement in a visual acuity task did not reach statistical significance. MRI scans were unchanged throughout the study. None of the patients had a clinical relapse in the trial, and measures of cognition and disability were stable. There were no serious adverse events, although clemastine was associated with significant fatigue as expected for a sedative drug (*p* = 0.017).

*Comment.* This small study showed that clemastine may lead to small improvements in P100 latencies on VEPs, and that these improvements may be sustained after stopping the drug. Any small functional improvements in visual acuity were not significant, and results were confounded by fatigue induced by clemastine. No direct evidence for CNS remyelination was presented. The trial had only 50 participants, imperfectly matched at baseline between the two study groups, and, therefore, lacked the power to detect small effect sizes. Furthermore, the trial lasted just 150 days, not long enough to measure any long-lasting effects. Therefore, although the observations reported are intriguing, further trials with more participants and better controls are warranted.

Green AJ et al (2017) Lancet 390: 2481–2489.

## Conclusion

Systematic, strategic drug repurposing is dependent on having robust, validated screening assays that bear relevance to the disease being targeted. These assays will usually be cell-based to allow upscaling and increased throughput, and care must be taken to measure meaningful outputs, particularly in neuropsychiatric diseases. Besides identifying potential therapeutics, these screens can also highlight new areas of molecular pathology for investigation, as shown in the PD and ZIKV studies. A greater understanding of cell pathology should enhance screening assays and iteratively accelerate drug development. Ultimately, however, large and expensive phase 3 clinical trials are still required to demonstrate drug efficacy against a specific disease in humans. The repurposing of drugs that are off-patent is unlikely to be lucrative for pharmaceutical companies, although adapting and developing these compounds may well be more fruitful than de novo drug design.

